# Unmasking Epstein-Barr Virus (EBV)-Positive Large B-Cell Lymphoma as an Underlying Trigger in a Post-COVID Hemophagocytic Lymphohistiocytosis Case

**DOI:** 10.7759/cureus.100374

**Published:** 2025-12-29

**Authors:** Dadmehr Yaghoubi, Jasmeet S Dhaliwal, Jose A Guerrero, Jacob Pilley, Nathan T VanderVeen

**Affiliations:** 1 Internal Medicine, University of California Los Angeles (UCLA), Los Angeles, USA; 2 Anaesthesiology, University of California Los Angeles (UCLA), Los Angeles, USA; 3 Pathology, West Los Angeles VA Medical Center, Los Angeles, USA; 4 Internal Medicine-Pediatrics, University of California Los Angeles (UCLA), Los Angeles, USA

**Keywords:** covid-19, epstein-barr virus, epstein-barr virus-positive diffuse large b-cell lymphoma, hemophagocytic lymphohistiocytosis (hlh), h-score, lymphoma-associated hemophagocytic syndrome, secondary hlh

## Abstract

Hemophagocytic lymphohistiocytosis (HLH) is a life-threatening hyperinflammatory syndrome caused by dysregulated activation of cytotoxic T cells and macrophages, most commonly triggered by infection or malignancy. We describe a 79-year-old male with a remote history of multiple malignancies, all in remission, who presented with persistent fevers and was found to have pancytopenia and transaminitis following a COVID-19 infection two weeks earlier. An elevated H-score prompted early bone marrow biopsy, which confirmed hemophagocytosis and revealed a smoldering Epstein-Barr virus (EBV)-positive large B-cell lymphoma as the likely underlying trigger. Initiation of dexamethasone monotherapy led to rapid clinical and laboratory improvement before definitive chemotherapy for lymphoma was considered. This case highlights the "threshold model" of HLH, in which compounding insults such as COVID-19 infection and occult EBV-positive lymphoma can breach the immunologic tipping point. It also underscores the importance of maintaining a high index of suspicion for rare but life-threatening conditions such as HLH. Prompt H-score calculation and bone marrow sampling can secure the diagnosis, uncover the underlying trigger, and expedite appropriate therapy in patients with HLH.

## Introduction

Hemophagocytic lymphohistiocytosis (HLH) is a potentially life-threatening hyperinflammatory syndrome driven by activation of cytotoxic T lymphocytes and macrophages, leading to excessive production of pro-inflammatory cytokines [1]. This maladaptive response has been associated with certain genetic variants, although triggers such as infection, malignancy, or underlying autoimmune conditions are typically required even in those with inherited familial HLH [2]. Classically, patients with inherited genetic mutations are considered to have primary HLH and commonly present in childhood. The first gene identified was *PRF1*, which encodes perforin, a pore-forming protein involved in cytotoxic granule-mediated target-cell lysis. Since then, numerous mutations, including *PRF1* deletions, have been associated with primary HLH [3]. Conversely, secondary HLH presents in older individuals in the absence of a known genetic variant and can result from similar triggering events as described above [4]. Among infectious causes, Epstein-Barr virus (EBV) has been most commonly associated with HLH [2]; however, emerging evidence suggests that COVID-19 infection can also act as a potent immune trigger [5-7].

Malignancy-associated HLH represents 28% of all secondary cases, of which approximately 76% are lymphoma-associated hemophagocytic syndrome (LAHS) [4]. Among hematologic neoplasms, EBV-driven lymphomas are classic culprits, although any high-grade lymphoid malignancy may incite macrophage overactivation [8].

Traditionally, HLH is recognized in the context of fulminant presentations - patients frequently exhibit shock, acute liver failure, and disseminated intravascular coagulation (DIC) [9]. However, early or "smoldering" forms of this syndrome may present more subtly, with persistent low-grade fever and cytopenias in the absence of hemodynamic or respiratory instability, which can lead to potential diagnostic delay.

We present a case of indolent EBV-positive large B-cell LAHS likely precipitated by a recent COVID-19 infection in a clinically stable patient, in whom prompt HLH evaluation and early bone marrow biopsy unmasked an underlying malignancy. We also discuss practical approaches for timely HLH identification and diagnosis after COVID-19 infection.

## Case presentation

This is a case of a 79-year-old male with a remote history of prostate cancer treated with prostatectomy and radiation; angioimmunoblastic T‑cell lymphoma treated with autologous peripheral blood stem cell transplant five years prior to presentation, which was complicated by T-cell large granular lymphocytic leukemia; and tonsillar squamous cell carcinoma, which was resected five years prior, presenting to the emergency room with several days of weakness and fever without prominent respiratory symptoms.

On presentation, the patient met sepsis criteria with a fever of 38.8 °C, a heart rate of 103 beats/min, and a respiratory rate of 28 breaths/min. Their blood pressure was 140/68 mmHg, and oxygen saturation was 98% on room air. COVID-19 PCR testing was positive. Chest radiograph obtained on admission showed no focal consolidation or interstitial infiltrates. Initial laboratory evaluation demonstrated pancytopenia (WBC of 3.55 x 10³/µL, Hgb of 12.0 g/dL, Plt of 101 x 10³/µL), elevated liver enzymes (AST of 201 U/L, ALT of 125 U/L, ALKP of 83 U/L), and acute kidney injury (creatinine of 1.30 mg/dL; baseline creatinine range: 0.8-0.9 mg/dL).

The patient received four doses of remdesivir in addition to fluid resuscitation; the fifth dose was held due to rising liver enzymes. Steroids were deferred given normal oxygen levels. By hospital day four, despite improvement in respiratory symptoms and negative COVID-19 cycle threshold, the patient developed worsening pancytopenia (WBC of 2.81 x 10³/µL; Hgb of 10.7 g/dL; Plt of 47 x 10³/µL) and markedly elevated liver enzymes (ALT of 262 U/L, AST of 601 U/L, ALKP of 138 U/L), with lactate dehydrogenase (LDH) of 987 U/L. Broad-spectrum antibiotics were initiated due to suspicion of secondary bacterial infection after COVID-19 without clinical improvement.

**Figure 1:**
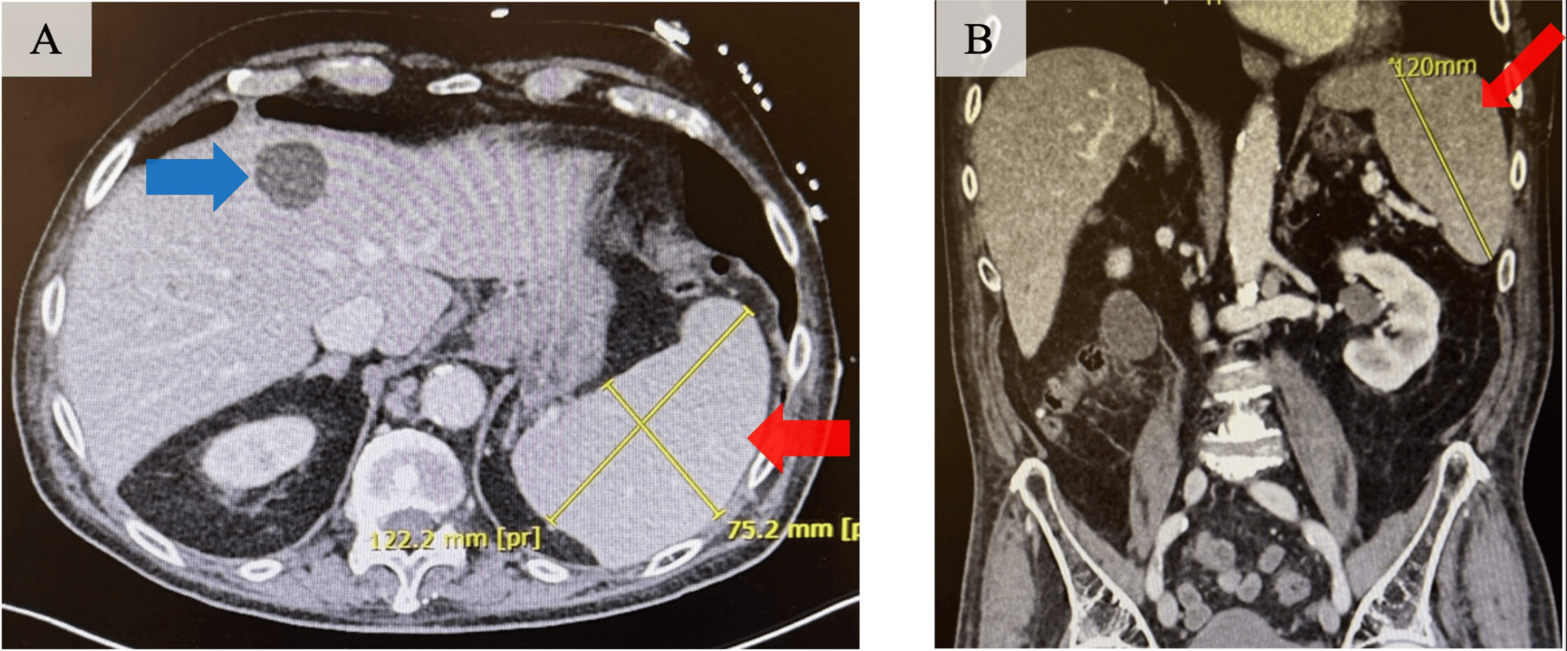
CT abdomen/pelvis with contrast (hospital day five) (A) Transverse image of the spleen and liver showing an enlarged spleen (red arrow) based on splenic index* of 1083, along with a stable hepatic hemangioma (blue arrow) present in previous imaging. (B) Coronal image of the abdomen showing splenic enlargement (red arrow). CT: Computed Tomography, *Splenic Index (SI) = spleen length × width × thickness Original clinical image; patient identifiers removed. Written informed consent for publication was obtained from the patient.

On hospital day five, further workup was done to evaluate for secondary bacterial infections, recurrence of malignancy, and drug-induced marrow suppression, causing persistent fevers and worsening pancytopenia. CT abdomen/pelvis demonstrated enlarged retrocrural lymph nodes and an enlarged spleen (Figures 1A-1B). Right upper quadrant ultrasound also showed hepatomegaly, non-specific gallbladder wall thickening, and a small amount of pericholecystic fluid. Empiric antibiotics for presumed acalculous cholecystitis were initiated based on the elevated alkaline phosphatase (ALKP) and gamma-glutamyl transferase (GGT) with imaging evidence of gall bladder thickening and pericholecystic fluid, though sonographic Murphy’s sign was negative and the patient denied abdominal pain. A hepatobiliary iminodiacetic acid (HIDA) scan was also scheduled to formally assess for acalculous cholecystitis, given diagnostic uncertainty.

**Figure 2:**
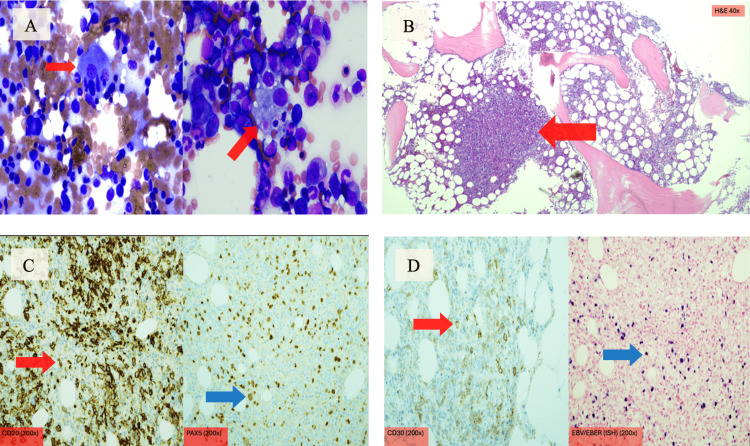
Bone marrow biopsy histopathology slides showing hemophagocytosis and EBV+ B-cell lymphoma Original clinical image; patient identifiers removed. Written informed consent for publication was obtained from the patient. (A) Macrophages with intracellular RBCs, lymphocytes, and neutrophils (red arrows - top left panel). (B) Lymphohistiocytic nodules within the marrow space (red arrow - top right panel). (C) CD20 (red arrow - bottom left panel) and PAX5 (blue arrow - bottom left panel) B-cell marker staining showing large scattered cells within the background of the lymphohistiocytic nodules. The large cell size can be appreciated well on the PAX5 stain. (D) Large cells stain positive for CD30 (red arrow - bottom right panel) and EBV (blue arrow - bottom right panel).

By hospital day seven, the patient remained hemodynamically stable but continued to have daily fevers, progressively worsening cytopenias (WBC of 2.50 x 10³/µL; Hgb of 8.0 g/dL; Plt of 23 x 10³/µL), and persistent liver enzyme elevation. At this point, the medical team had a high clinical suspicion for HLH, which prompted evaluation of a ferritin and fasting triglyceride level, which revealed hyperferritinemia (>1,650 ng/mL) and hypertriglyceridemia (345 mg/dL). Hematology was consulted for possible HLH versus infiltrative diseases, such as hematologic malignancies, Langerhans Cell histiocytosis, or Castleman disease. A bone marrow biopsy on hospital day eight revealed hemophagocytosis (Figure 2A). Ferritin was measured at 6,645 ng/mL on hospital day nine, and soluble interleukin-2 receptor (sCD25) eventually revealed an elevated level of 64,525 pg/mL (normal range: 532-1891 pg/mL).

**Figure 3:**
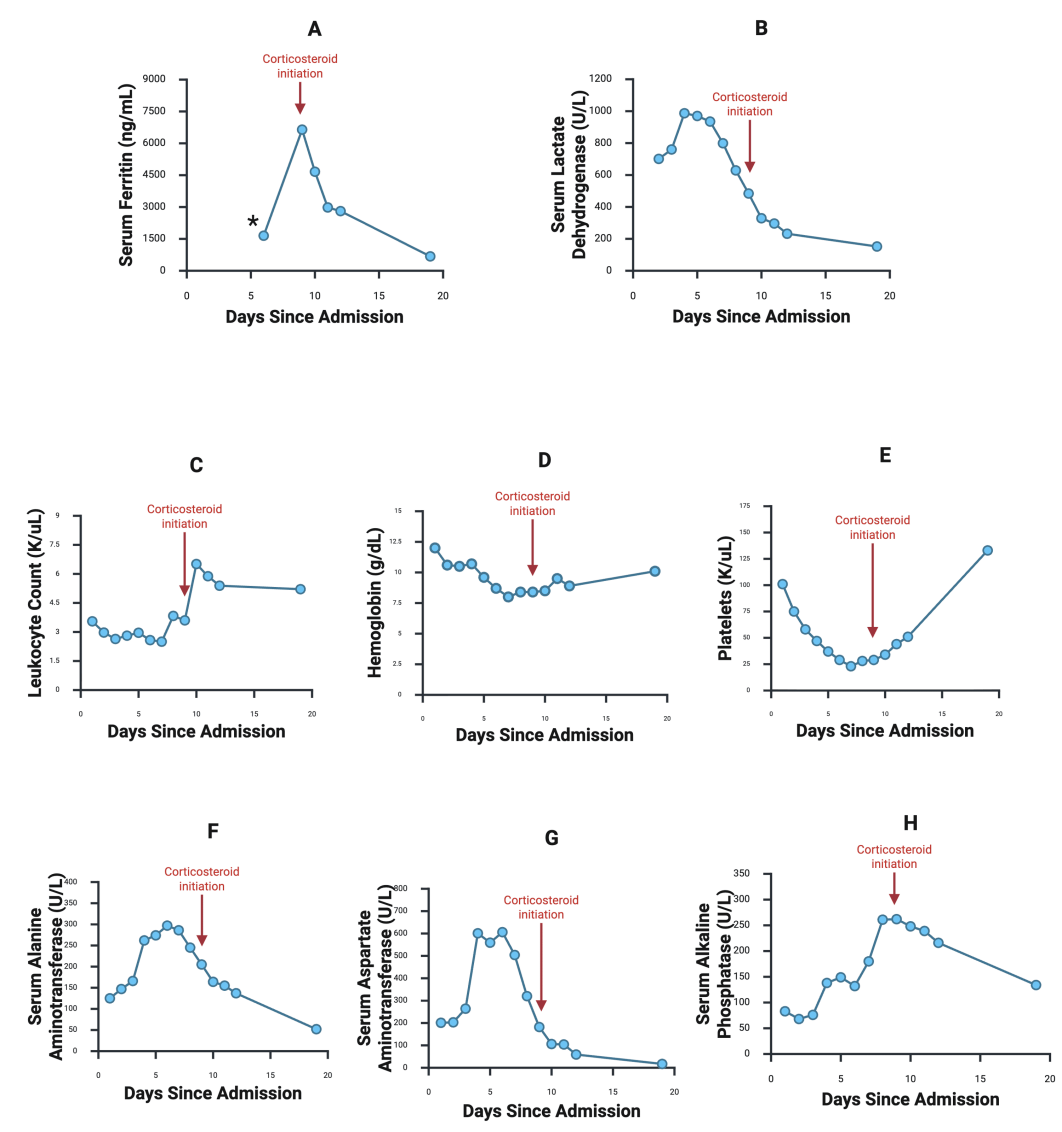
Serum laboratory trends Red arrows display treatment initiation with corticosteroids on day nine. (A) Ferritin, (B) lactate dehydrogenase, (C) leukocyte count, (D) hemoglobin, (E) platelets, (F) alanine aminotransferase, (G) aspartate aminotransferase, and (H) alkaline phosphatase *First ferritin measurement was “>1,650 ng/mL” based on laboratory standards for the initial sample.

To formally evaluate the patient’s risk of HLH, an H-score of 253 was calculated, reflecting a >99% probability of HLH (Table 1). Due to the patient’s clinical stability and moderate severity of HLH, etoposide was deferred, and dexamethasone was initiated on hospital day nine. Eventually, the patient fulfilled each of the eight HLH 2024 criteria: persistent fever, splenomegaly, cytopenias, hyperferritinemia, hypertriglyceridemia, hemophagocytosis on bone marrow biopsy, and elevated sCD25. After treatment initiation, the patient’s fevers resolved, and laboratory parameters began to normalize. Antibiotics were discontinued, and the HIDA scan was deferred, given the lack of clinical symptoms of cholecystitis. The patient was discharged on hospital day 12 on a steroid taper. Upon follow-up one week later, their lab work showed a downtrending ferritin (677 ng/mL), normalization of liver enzymes, and return of this patient’s pancytopenia back to their baseline (Figure 3, Table 2).

**Table 1:** H-score calculation for reactive hemophagocytic syndrome probability AST: Aspartate Aminotransferase; CTAP: Computed Tomography Abdomen-Pelvis; US: Ultrasound

Parameter	Patient Value	Points
Known immunosuppression	No	0
Temperature ≥ 38.4 °C	Persistent fevers after COVID-19 clearance	33
Organomegaly	Hepatomegaly on US, Splenomegaly on CTAP (Splenic index >480)	38
Number of cytopenias (≥2 lineages)	Pancytopenia	34
Ferritin ≥ 2,000 ng/mL	6,645 ng/mL (peak)	50
Triglycerides ≥ 133 mg/dL (1.5 mmol/L)	345 mg/dL	44
Fibrinogen ≤ 250 mg/dL	284 mg/dL (on day 9)	0
AST > 30 U/L	201 U/L (admission); 601 U/L (peak)	19
Hemophagocytosis on bone marrow biopsy	Present	35
Total H-Score	Probability of HLH: >99%	253

**Table 2:** Serial serum laboratory results during hospitalization and post discharge with corresponding reference ranges D#: hospital day; Steroid Start: The day corticosteroid course was initiated; Outpt: Outpatient follow-up labs ALT: alanine transaminase; AST: aspartate aminotransferase; ALKP: alkaline phosphatase; LDH: lactate dehydrogenase

Parameter (Units)	Reference Range (Units)	D1	D2	D3	D4	D5	D6	D7	D8	D9 (Steroid Start)	D10	D11	D12	D19 (Outpt)
WBC (×10³/µL)	4.5–11.0 (×10³/µL)	3.55	2.97	2.64	2.81	2.96	2.58	2.5	3.83	3.6	6.51	5.88	5.39	5.21
Hgb (g/dL)	13.3–17.7 (g/dL)	12	10.6	10.5	10.7	9.6	8.7	8	8.4	8.4	8.5	9.5	8.9	10.1
Platelets (×10³/µL)	150–440 (×10³/µL)	101	75	58	47	37	29	23	28	29	34	44	51	133
Ferritin (ng/mL)	22–322 (ng/mL)	—	—	—	—	—	>1650	—	—	6645	4662	2981	2808	677
Triglycerides (mg/dL)	<150 (mg/dL)	—	—	—	—	—	345	—	—	—	289	—	320	—
Fibrinogen (mg/dL)	253–390 (mg/dL)	—	—	458	—	—	—	—	—	284	250	266	259	—
AST (U/L)	13-35 (U/L)	201	203	264	601	559	606	504	320	182	106	104	59	17
ALT (U/L)	7-45 (U/L)	125	147	166	262	274	297	286	245	205	164	155	137	52
ALKP (U/L)	33-94 (U/L)	83	68	76	138	149	132	180	261	262	248	239	216	134
LDH (U/L)	87-271 (U/L)	—	701	760	987	970	935	799	629	484	329	296	232	152

Upon final analysis of the bone marrow biopsy, the patient was found to have hypercellular marrow, EBV+ large B-cell lymphoma with atypical lymphohistiocytic infiltrates with occasional hemophagocytosis (Figures 2A-2D).

## Discussion

Pathophysiology

**Figure 4:**
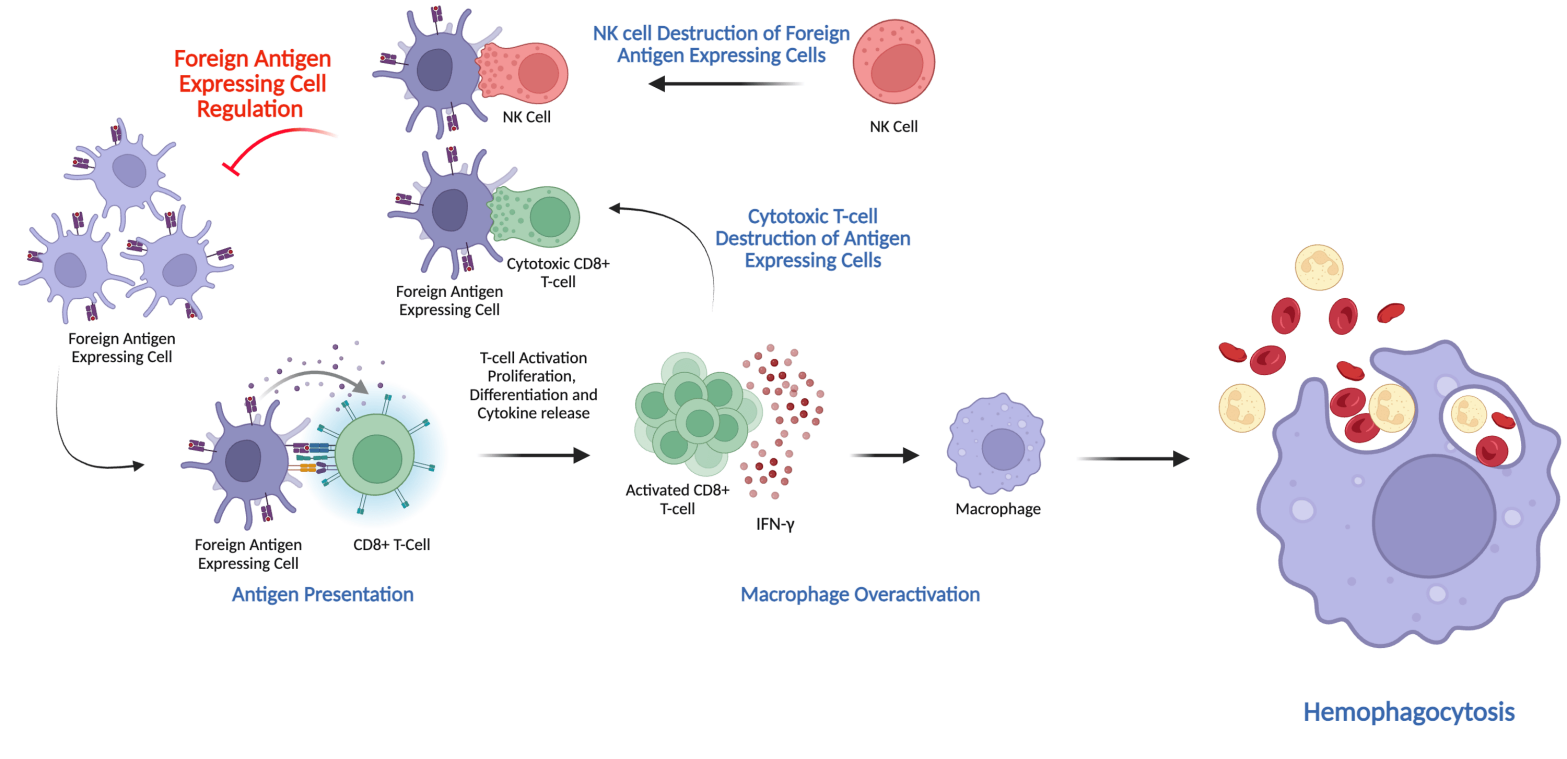
Schematic of the immune response to a foreign antigen expressing cell (FAEC)—which may represent either a virally infected cell (e.g., EBV+) or a malignant cell displaying aberrant antigens—with depiction of immune overactivation that drives familial hemophagocytic lymphohistiocytosis (HLH) Under homeostatic conditions (top left), natural killer (NK) cells and cytotoxic CD8+ T-cells eliminate over-activated FAECs to constrain antigen presentation. Persistent antigen presentation (bottom left) engages more CD8+ T-cells via MHC I, leading to their activation, proliferation, and IFN-γ secretion (center). Elevated IFN-γ levels hyperactivate macrophages, which then phagocytose erythrocytes, leukocytes, and platelets (right)—the hallmark hemophagocytosis seen in HLH. Created by authors in BioRender (https://BioRender.com/geldz7v) Schematic based on concept adapted from Jordan [2]

HLH is defined as a non-malignant multi-system inflammatory syndrome caused by defects in inhibitory cross-talk between the innate and adaptive immune system [1]. After activation by foreign antigen-expressing cells (FAECs) - which may include virally infected cells or malignant cells displaying aberrant antigens - through their major histocompatibility complex class I (MHC-I), CD8+ T cells differentiate, proliferate, release inflammatory cytokines, and develop their cytotoxic function. Laboratory mice models of primary HLH have shown the inhibitory and cytotoxic effects of activated T cells along with natural killer (NK) cells on the FAEC population, presenting that the original antigen is crucial in regulating its “re-presentation” to other CD8+ T cells, avoiding excessive release of interferon gamma (IFN-γ) [4,10]. Specifically, perforin/granzyme pathways are involved in this cytolytic ability of NK and T-cells [11,12]. In the pathological state of HLH, any loss in this inhibitory pathway causes over-stimulation of CD8+ T-cells, IFN-γ production, and over-activation of downstream macrophages. This results in the uncontrolled inflammatory response seen in HLH, leading to phagocytosis of other blood cell lines, thus resulting in progressive cytopenia [2]. This process is summarized schematically in Figure 4, which illustrates how impaired cytotoxic regulation leads to macrophage overactivation and hemophagocytosis in familial HLH. It is postulated that the pathophysiology of LAHS is due to the inability of the immune system to clear the malignant lymphocyte, leading to prolonged immune “synapse” formation and overstimulation of CTLs. In this case, we hypothesize that EBV-positive malignant large B-cells evaded immune elimination or overwhelmed existing immune regulatory mechanisms, with concurrent COVID-19 infection contributing to excess T-cell activation and cytokine release.

COVID-19 has been reported to cause HLH [5-7,13] and likely acted as an additional immune trigger for this patient, although definitive causality cannot be established. COVID-19 infection is associated with a significant cytokine storm fueled by both innate and adaptive immune systems. Following entry into the cell by SARS-CoV-2 through the angiotensin-converting enzyme 2 (ACE2) receptor in the lung parenchyma, toll-like receptors and NOD-like receptors sense viral components and activate transcription factors such as NF-κB, promoting the expression of pro-inflammatory cytokines such as IL-6, IL-1β, and TNF-α. There is currently a lack of consensus about why this cytokine storm could cause HLH; however, studies have shown that elevated IL-6 levels impair NK-cell cytotoxicity by downregulating perforin and granzyme B, mimicking known genetic mutations in primary HLH [14], and thus delaying early viral clearance and permitting sustained antigen presentation by FAECs. The persisting antigen load drives unchecked CD8+ T-cell activation and massive IFN-γ secretion, which in turn hyperactivates macrophages. Additionally, the characteristic expansion of T-cells with high CD38 signal on flow cytometry is a key feature that differentiates HLH from sepsis but has also been observed in the context of COVID-19 infections [15,16], suggesting similar mechanistic pathways.

These findings align with the “threshold model” of HLH, which proposes that HLH occurs when the cumulative effect of multiple immunologic stimuli-genetic, infectious, or malignant exceeds a critical threshold for immune activation [17]. In this patient, the combination of persistent antigenic stimulation from EBV-positive large B-cell lymphoma and recent COVID-19 infection likely acted synergistically to surpass that threshold, triggering sustained cytotoxic T-cell activation and macrophage-driven inflammation.

Diagnosis

HLH is frequently misdiagnosed as sepsis, as initially occurred in our patient. The patient received an extensive evaluation for new secondary infections and was started on empiric antimicrobials, initially targeting COVID-19 infection and subsequently acalculous cholecystitis given his septic presentation. However, when the patient clinically worsened despite therapy, the differential diagnosis was revisited. Typical “red flag” features of infection - seizures, altered mental status, respiratory failure, or severe liver injury - were absent, despite their common occurrence in critically ill HLH cases, which made arriving at the ultimate diagnosis challenging [9].

New diagnostic criteria were developed in 2024 for the diagnosis of HLH, which include persistent fevers, splenomegaly, cytopenias, hyperferritinemia, hypertriglyceridemia, hemophagocytosis on bone marrow biopsy, and elevated sCD25 [18]. Because some HLH criteria (e.g., sCD25) often delay diagnostic confirmation, the H-score - a validated point system that has been shown to have 100% sensitivity and 94% specificity if above the 168 score - can quickly and effectively stratify risk of HLH [19]. In recent studies, higher H-scores in critically ill patients with COVID-19 have been associated with higher HLH severity and increased mortality [6]. Therefore, early risk stratification and evaluation for HLH through the H-score is of utmost importance, especially in post-COVID-19 patients with unexplained fever and pancytopenia, as they may prompt the timely initiation of corticosteroids or other immunomodulators.

After confirming HLH, prompt identification of possible underlying triggers, especially malignancy, becomes paramount. In our case, bone marrow biopsy played a dual purpose: confirming hemophagocytosis consistent with HLH and unexpectedly identifying EBV-positive large B-cell lymphoma, thus revealing the core diagnosis and directly informing definitive management. Clinicians should maintain a low threshold for early bone marrow sampling to avoid diagnostic delays and ensure targeted therapy initiation, particularly in patients at risk of malignancy. Additionally, an elevated sCD25-to-ferritin ratio ≥ 2.0 has demonstrated predictive value for lymphoma-associated HLH [20], providing an additional clue when initial workup remains inconclusive.

Treatment

In the case of LAHS, the general treatment involves early immunosuppression with corticosteroids and/or etoposide to avoid further organ failure from the cytokine storm. The choice of adding etoposide is dependent on the severity of HLH, CNS involvement, and multi-organ failure since corticosteroid therapy alone is sufficient in moderate secondary HLH. This is promptly followed by cancer-directed therapies to eliminate the immune trigger [4].

This case underscores the potential adequacy of corticosteroid monotherapy as first-line treatment for HLH in clinically stable patients when the trigger is not yet known, but infection has been ruled out. Given the risks associated with cytotoxic agents such as etoposide, a stepwise escalation based on clinical severity may be appropriate in milder cases. However, clinicians must remain vigilant and pursue further etiologic workup - including malignancy screening - even after initial improvement.

## Conclusions

This case highlights that HLH can manifest insidiously without overt multi-organ failure. In clinically stable, post-COVID-19 patients with persistent fevers and cytopenias, early use of the H-score and prompt bone marrow biopsy can both confirm HLH and potentially unmask an occult trigger such as malignancy. Although our patient appeared clinically stable compared to classic HLH cases, they ultimately fulfilled all eight 2024 HLH criteria. These observations reinforce the “threshold model” of HLH, whereby COVID-19 and EBV-positive large B-cell lymphoma synergistically breached the immunologic tipping point. Maintaining a low diagnostic threshold in similar scenarios could shorten time to treatment initiation and improve outcomes in smoldering HLH.
